# Comparing passive measures of fatigue-like behavior in mice

**DOI:** 10.1038/s41598-018-32654-1

**Published:** 2018-09-24

**Authors:** Brian S. Wolff, Sumiyya A. Raheem, Leorey N. Saligan

**Affiliations:** National Institute of Nursing Research, National Institutes of Health, Bethesda, Maryland USA

## Abstract

Fatigue is a very common and costly symptom associated with various diseases and disorders. Nonetheless, understanding the pathobiology and developing of therapies for fatigue have been difficult, partly because of a lack of consensus on the measures to phenotype this behavior, both in clinical settings and in animal studies. Here, we describe a fatigue-like behavior induced in mice by abdominal irradiation and compare three different methods of measuring changes in physical activity over time: running wheels, video home cage monitoring, and telemetry. These methods collect data passively and continuously, requiring no disruption of animals’ normal home cage behavior. In our experiments, all three methods reported a fatigue-like behavior, exhibited by a reduction in physical activity following abdominal irradiation. Video tracking showed the largest fatigue effect size (Cohen’s D = 1.78) over four days of monitoring, and was the only method showing a significant decrease in activity during the light period. Telemetry and running wheels showed a similar effect size (D = 1.68 and 1.65, respectively), but running wheels showed different circadian patterns of physical activity. In addition, we conducted rotarod and inverted grid suspension tests, which suggested that fatigue-like behavior was not the result of gross motor abnormalities.

## Introduction

Fatigue is a common, bothersome symptom that has a very high negative impact on a person’s quality of life and significant repercussions on both direct and indirect health economic outcomes^1^. Fatigue is associated with a wide range of disorders such as cancer^2^, stroke^3^, and Parkinson’s disease^4^, and also frequently with other concurrent symptoms such as insomnia and depression^5^. Unfortunately, the wide range of associated factors has made fatigue a difficult concept to study or even define.

Although fatigue is considered to be a multidimensional behavior, it is mostly manifested as a decline in physical function and daily activity^6^. Much of the published work on mouse models of fatigue focuses on cancer-related fatigue, which can be induced by tumor growth^7–10^, chemotherapy^10–15^, or radiation^10,16,17^. Other published studies induce fatigue using lipopolysaccharide^18^ or exercise^19,20^. In all studies, fatigue was measured as a decline in physical activity following fatigue induction.

In clinical studies, fatigue is typically determined by self-reports and questionnaires, and rarely using behavioral or physical measurements^2^. In contrast, with preclinical mouse models investigators have published quite a few behavioral measures of physical activity to measure fatigue, including: running wheels^7–9,11,12,14–17,20,21^; home cage monitoring using video^7,8,18^, telemetry^11^, or cages with light beams/sensors^20^; treadmills^10,19,21^; forced swim^8,9^; and open field^12^. However, there are no standards for which methods should be considered reliable indicators of fatigue. So as an initial effort, this study was conducted to compare measures that assess physical activity and physical function to identify the most effective methods to measure fatigue-like behavior in mice. Identification of the most effective method may prove valuable to both the mechanistic study of fatigue and development of treatments.

In this study, we induced a fatigue-like change in behavior using targeted abdominal irradiation, an animal model of fatigue developed to mimic localized radiation therapy for non-metastatic prostate cancer patients. We chose this model because (1) it is not caused by a specific disease, (2) it induces fatigue without causing any direct damage to the brain or other major organ systems, (3) it generates a trajectory of fatigue that resolves to baseline values, and (4) it can potentially be translated into a repeated stress model or an inflammatory model of fatigue. Because multiple environmental conditions influence behavior of mice^22^ and it was important to ensure wide utility of the selected fatigue measures, methods were selected that would allow spontaneous behavior of the animals. We tested three different methods of measuring fatigue, all of which monitored physical activity passively. The methods tested did not require experimenter interaction with the mice nor did they disrupt the normal daily activity of the mice.

## Results

**Figure 1:**
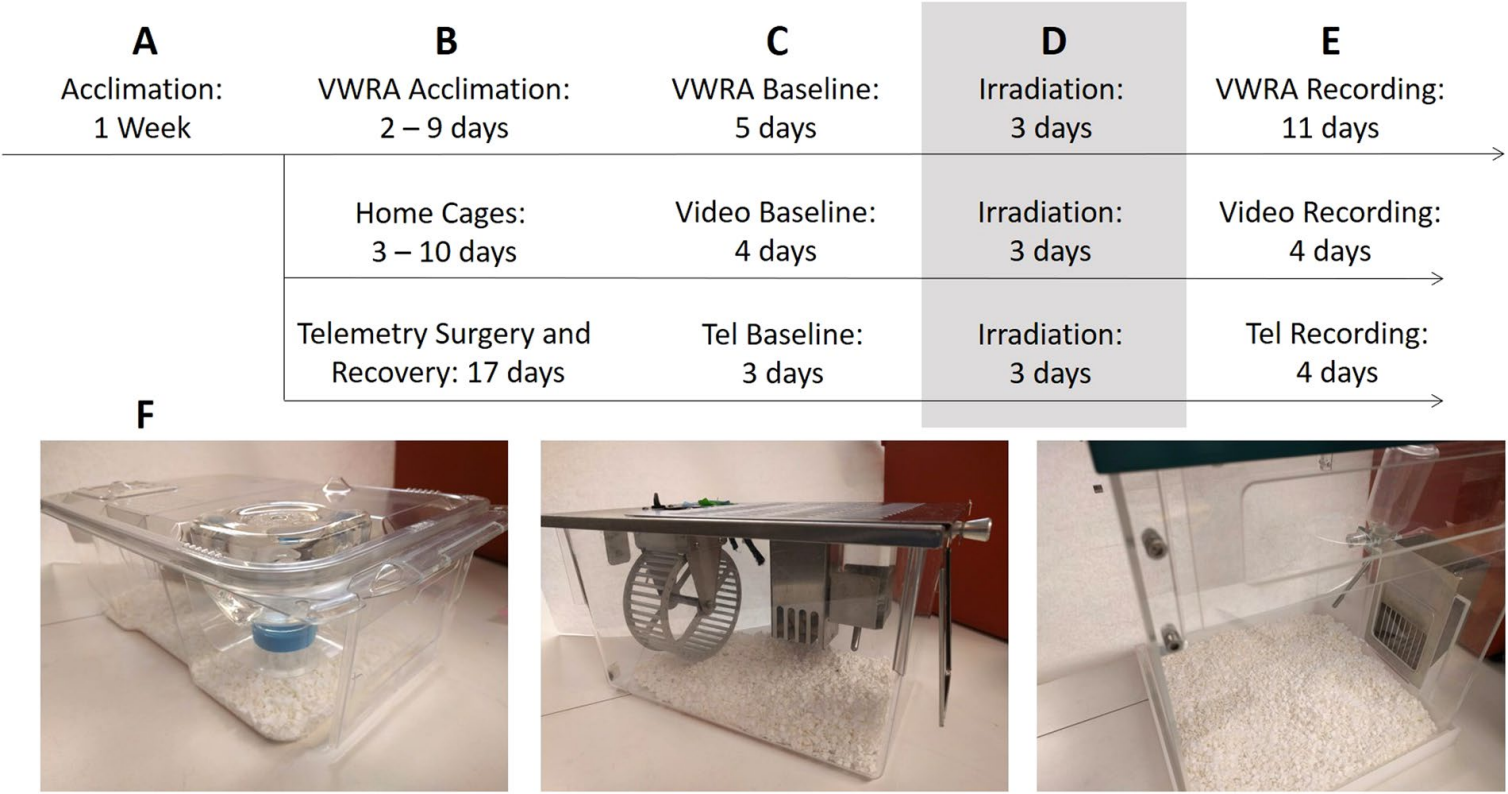
Experiment design. (**A**) After arriving in the animal facility, mice were allowed to acclimate for one week. During this time, they received tail tattoos and were gently handled for three days. (**B**) Mice then were split into three groups: the VWRA group (n = 63) acclimated to running wheel cages, the video group (n = 20) remained in home cages, and the telemetry group (n = 20) remained in home cages after surgical implantation of telemetry devices. (**C**) Prior to irradiation, 3–5 days of data collection constituted the baseline level of physical activity. (**D**) Mice were irradiated for three consecutive days, receiving 8 Gy per day to the thighs and lower abdomen; regardless of group, all mice were housed in standard home cages during this time. (**E**) Post-irradiation data collection began the day after irradiation concluded. (**F**) Pictures of the three different cages: standard home cages (left), running wheel cages (center), and PhenoTyper video home cages (right).

Mice received three consecutive days of irradiation, with a dose of 8 Gy per day to a restricted area in the lower abdomen. Figure 1 describes the timeline of activities included in this experiment. The wheel running group began with 72 mice, but eight died during the study and three did not use the running wheels. The video and monitoring group began with 20 mice, but one died during the irradiation procedure. The telemetry group began with 22 mice, but two died during the irradiation procedure and one was removed from the study due to health concerns. The three days of irradiation will be referred to as days 0–3.

### Total Distance

#### Voluntary Wheel Running

**Figure 2:**
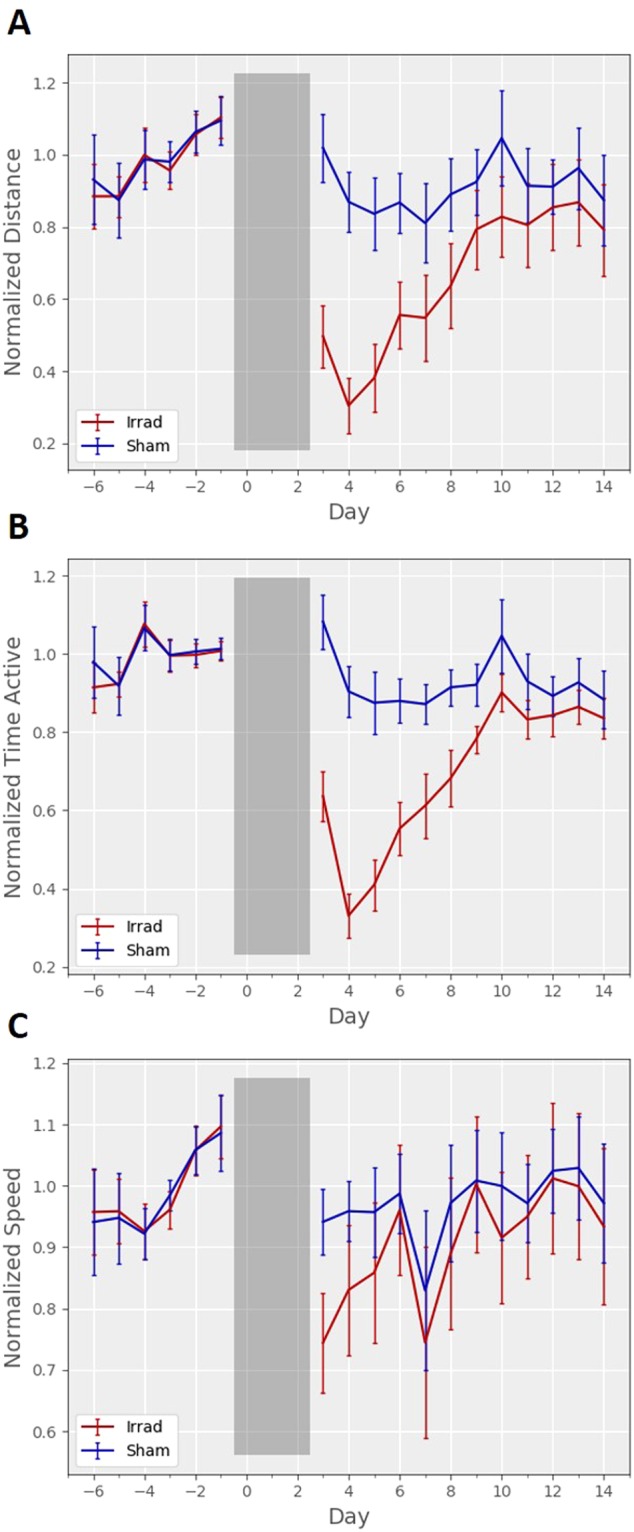
Daily wheel running activity. (**A**) Daily total distances run on the wheels showed a significant difference between groups after irradiation (F_1,61_ = 15.83, p < 10^−3^). (**B**) “Time active,” the number of minutes during which a mouse used the running wheel, wheels showed a significant difference between groups after irradiation (F_1,61_ = 27.29, p < 10^−5^). (**C**) Average (mean) speed measurements showed higher variability and did not show a significant difference between groups after irradiation (F_1,61_ = 3.54, p = 0.065). Data are normalized to the mean total across the five days before irradiation, and error bars are 95% confidence intervals.

At baseline, mice ran an average of 8.76 ± 2.92 km per day (mean ± standard deviation). Average daily running wheel distances (Fig. 2A) across all mice were significantly reduced for six days after irradiation (p < 0.05, one-tailed t-test with Holm-Bonferroni correction), similar to our previous report^16^. We also discovered a potentially more relevant measure from the running wheel data, “time active,” which is the number of minutes during which the mouse used the wheel. Compared to distance, this measure (Fig. 2B) shows a nearly proportional and identical decrease in activity (with a trough between 36% and 37% of baseline), however, it shows lower variability. Daily wheel time totals were significantly reduced for seven days after irradiation (p < 0.05, one-tailed t-test with Holm-Bonferroni correction). Distance measurements shown in Fig. 2A showed a higher variability than the time active measurements in Fig. 2B due to highly variable speeds, which are plotted in Fig. 2C. Unlike with distance (F_1,61_ = 15.83, p < 10^−3^) and wheel time (F_1,61_ = 27.29, p < 10^−5^), there was no significant difference in speed between groups after irradiation (F_1,61_ = 3.54, p = 0.065) as determined by a two-way linear mixed-model ANOVA.

#### Video Monitoring and Telemetry

**Figure 3:**
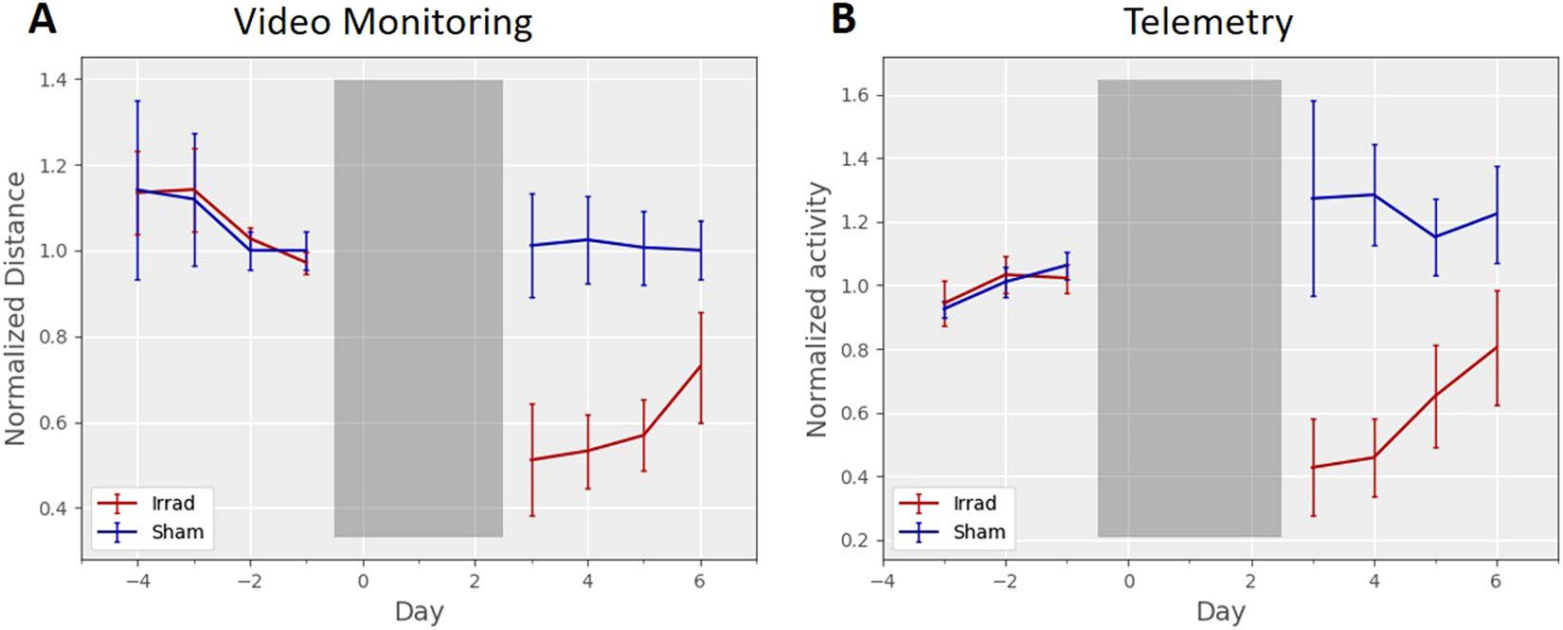
Daily home cage activity. (**A**) Video measurements of daily distance travelled in home cages monitored 24-hours per day were significantly lower in the irradiated group after irradiation (F_1,17_ = 64.75, p < 10^−6^). (**B**) Telemetry measurements of daily activity in home cages monitored 24-hours per day were significantly lower in the irradiated group after irradiation (F_1,17_ = 39.90, p < 10^−5^). Data are normalized to the average total across the two days before irradiation, and error bars are 95% confidence intervals.

At baseline in the video tracking measurements, mice moved on average 414 ± 206 meters per day (mean ± standard deviation). A two-way linear mixed-model ANOVA determined that both video tracking (F_1,17_ = 64.75, p < 10^−6^) and telemetry measurements (F_1,17_ = 39.90, p < 10^−5^) showed a significant difference between groups in distances travelled after irradiation (Fig. 3), a difference that was significant on all four days after irradiation (p < 0.05, one-tailed t-test with Holm-Bonferroni correction). With video tracking (Fig. 3A), the baseline recording became stable after two days, with daily distance totals becoming similar from day to day. These findings of stability contrasted with running wheel distances, which can take a week or more to stabilize^23–25^. However, while the telemetry data (Fig. 3B) showed a significant difference between groups on all four post-irradiation days, the average daily distances were less consistent from day to day. The daily activity measured by telemetry was drifting upwards during the baseline recording period, and activity levels for the sham group seemed to continue their upward drift; these mice showed 20–30% more activity after sham irradiation than they showed at baseline.

#### Comparison

Comparing the plots in Figs 2 and 3, running wheels show the lowest activity on day 2, while video or telemetry monitoring show lowest activity on day 1. We believe this can be explained by cage transfers causing one day of elevated running wheel activity. For example, one week after completion of irradiation (day 10), the mice were temporarily taken out of the running wheel cage for cleaning. In the plots in Fig. 2, and that night there was a corresponding elevation of running wheel activity. We think it is likely that the cage transfer for irradiation also caused an elevation in running wheel totals on day 3, and that this may temporarily counteract the fatigue behavior induced by radiation.

### Individual Variability of Fatigue Behavior

#### Voluntary Wheel Running

**Figure 4:**
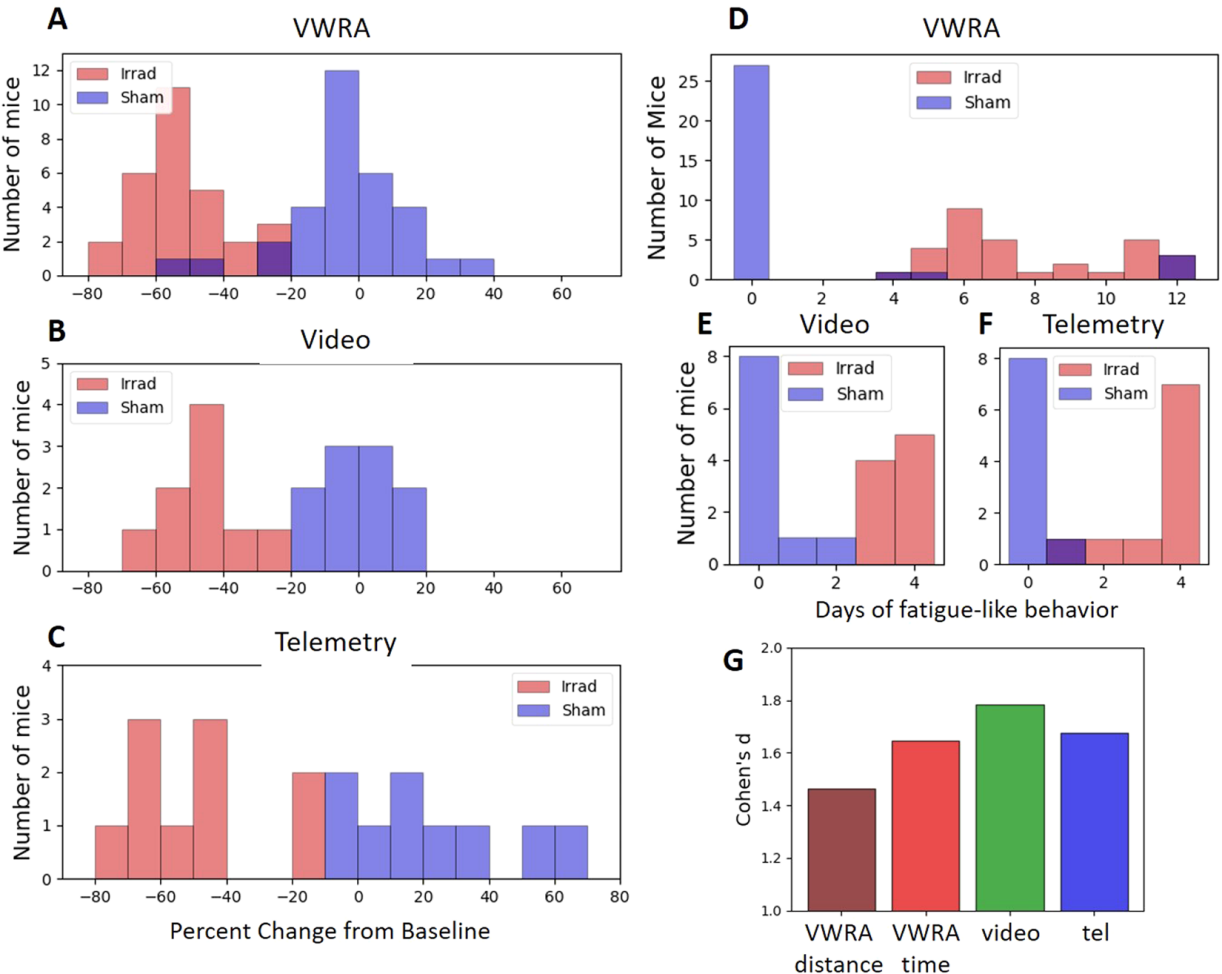
Fatigue histograms. (**A**–**C**) Histogram of the change in running wheel (**A**), video home cage (**B**) or telemetry home cage (**C**) activity for all mice, comparing in the four days after irradiation/sham to the three or four days prior. (**D**–**F**) Number of consecutive days of “fatigue-like” behavior, defined as activity below 80% of baseline. Running wheels show a few sham animals with a prominent “fatigue” phenotype. For all plots in (**A**–**F**), the two groups have significantly different distributions (two-sample Kolmogorov-Smirnov test, p < 10^−4^ for all comparisons). (**G**) Effect size for the different methods of measuring fatigue-like behavior. Video monitoring shows the largest effect. For running wheels, distance measures show a smaller effect than the time active.

We compared wheel running activity in the first four post-irradiation days relative to baseline for each individual animal (Fig. 4A). When measuring ‘time active’ in running wheels, the activity changes in the sham group showed a wide distribution centered near zero, and the irradiated group showed a slightly narrower distribution in which all irradiated mice had a reduction in activity relative to baseline. Although the irradiated and sham groups clearly showed different distributions, the small overlap showed that running wheels could not perfectly separate the irradiated and sham groups using the four-day total time active.

#### Home Cage Monitoring and Telemetry

Activity measured using video tracking (Fig. 4B) showed narrower distributions, albeit in a smaller sample size. With video tracking, there was no overlap between the distributions, meaning every irradiated mouse showed a greater reduction in activity than any sham mouse. While the same is true for the telemetry data (Fig. 4C), the telemetry data did not appear to be normally distributed. While the sample size is too small for an appropriate statistical test of normality, it may be that the sample size we used is too small to accurately describe the true population distribution.

### Duration of Fatigue-like Behavior

To assess the duration of behavioral changes in individual mice, we sought to define a simple threshold below which behavior would be considered “fatigue-like.” We chose a 20% reduction in activity for this threshold because all irradiated animals showed at least a 20% change in Fig. 4A,B. To reduce the impact of single-day fluctuations in running wheel totals (which may be caused by, among other things, cleaning of cages), we considered the fatigue to “end” only if activity was >80% of baseline for two or more consecutive days. The results of this analysis are plotted in Fig. 4D–F.

#### Voluntary Wheel Running

Running wheel recordings show a wide range of fatigue durations in irradiated mice. Of the 32 mice in the sham group, three sham mice appeared “fatigued” (as defined above), for the entire duration of the recording. An additional two mice in the sham groups exhibited the fatigue-like behavior for 4 or 5 days. Together, these five mice could be considered “false positives” for our simple thresholding method of detecting “fatigue” with running wheels. There were no corresponding “false negatives;” though, all 31 irradiated mice showed the fatigue-like behavior for at least four days.

#### Home Cage Monitoring and Telemetry

Although the video recordings were shorter in duration, the group distributions of duration of fatigue-like behavior (defined above) did not overlap. From video recordings, all nine irradiated mice in that testing group showed fatigue-like behavior for three or more days, while all sham mice showed fatigue-like behavior two or fewer days. Of the mice with telemetry implants, seven of the ten irradiated mice showed fatigue-like behavior for all four days of post-irradiation recording, and eight of the nine sham mice showed no fatigue-like behavior. However, the groups did not separate perfectly, because one mouse from each group showed a single day of fatigue-like behavior.

### Effect sizes

To assess the overall effectiveness of the methods of measurement, we calculated the effect sizes using Cohen’s d (Fig. 4G). As expected, running wheels showed the smallest effect size when measuring raw distances (d = 1.45), but improved when measuring time spent on the wheels (d = 1.64). Telemetry (d = 1.67) performed similarly to running wheel data using the ‘time active’ variable (d = 1.64), while video tracking showed the largest effect size (d = 1.75).

### Circadian Rhythm and Activity Patterns

**Figure 5:**
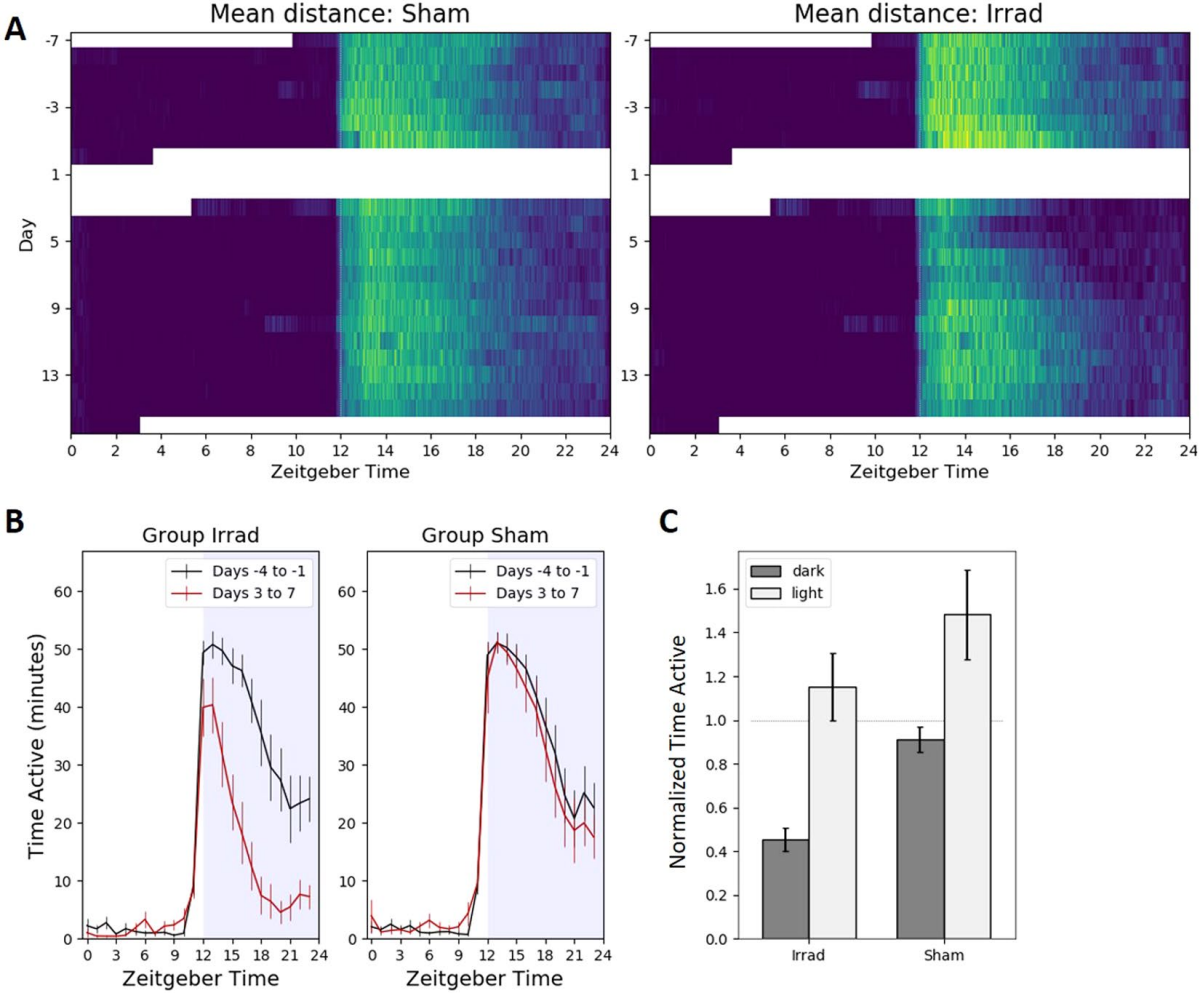
Circadian running wheel activity patterns. Zeitgeber time = 0 when the lights are turned on at 6am. All error bars are 95% confidence intervals. (**A**) Color-coded activity for each minute of recording, with brighter colors corresponding to more running wheel activity. (**B**) Running wheel activity averaged for every hour across four days of baseline and four days of post-irradiation recording. After irradiation, the irradiated group ran significantly less during the dark (one-tailed Bonferroni-corrected paired t-test, t_30_ = 11.69, p < 10^−18^) but not during the light (t_30_ = 1.08, p = 0.29). (**C**) Running wheel activity averaged for the 12-hour light period and the 12-hour dark period across four days of baseline and four days of post-irradiation recording. There was a significant effect of irradiation (two-way ANOVA, F_1,126_ = 35.98, p < 10^−7^), but no significant interaction between irradiation and time period (F_1,126_ = 0.95, p = 0.3). Bonferroni-corrected p-values for two-tailed t-tests: Irrad dark vs light: t_61_ = 8.70, p < 10^−11^. Sham dark vs light: t_61_ = 5.37, p < 10^−4^. Dark, irrad vs sham: t_61_ = 11.66, p = 10^−15^. Day, irrad vs sham: t_61_ = 2.17, p = 0.14.

It is well-established that mice show a pronounced circadian pattern in their daily physical activity, with more activity during the dark period than the light period of a 12:12 hour light-dark cycle. The images in Fig. 5 show the distribution of activity across the whole recording time, with the light period being from 6am/ZT 0 to 6 pm/ZT12.

#### Voluntary Wheel Running

As expected, almost all running wheel activity occurred during the dark period, with brief bursts in activity during the light period immediately after being transferred into a new cage (for cage cleaning). Running wheel activity on all days began just before the onset of darkness. On the second day after irradiation, when the fatigue-like behavior becomes most apparent (see Fig. 2A,B), the sham animals appear normal, but the irradiated animals stop running after only a few hours, possibly because they get tired sooner. Each day thereafter, the duration of nightly running activity slowly extended until it reached levels similar to baseline (Fig. 5A). Figure 5B shows average hourly activity over the four-day period immediately after irradiation, which is disproportionately reduced in the later hours of night. Activity during the dark period was significantly decreased after irradiation (p < 10^−15^), and light period activity, sparse as it was (Fig. 5A shows it is mostly during the first day), showed a marginally significant decrease as well (p = 0.49). A two-way ANOVA showed a significant effect of irradiation (F_1,126_ = 35.98, p < 10^−7^), but no significant interaction between irradiation and time period (F_1,126_ = 0.95, p = 0.3).

#### Home Cage Monitoring and Telemetry

**Figure 6:**
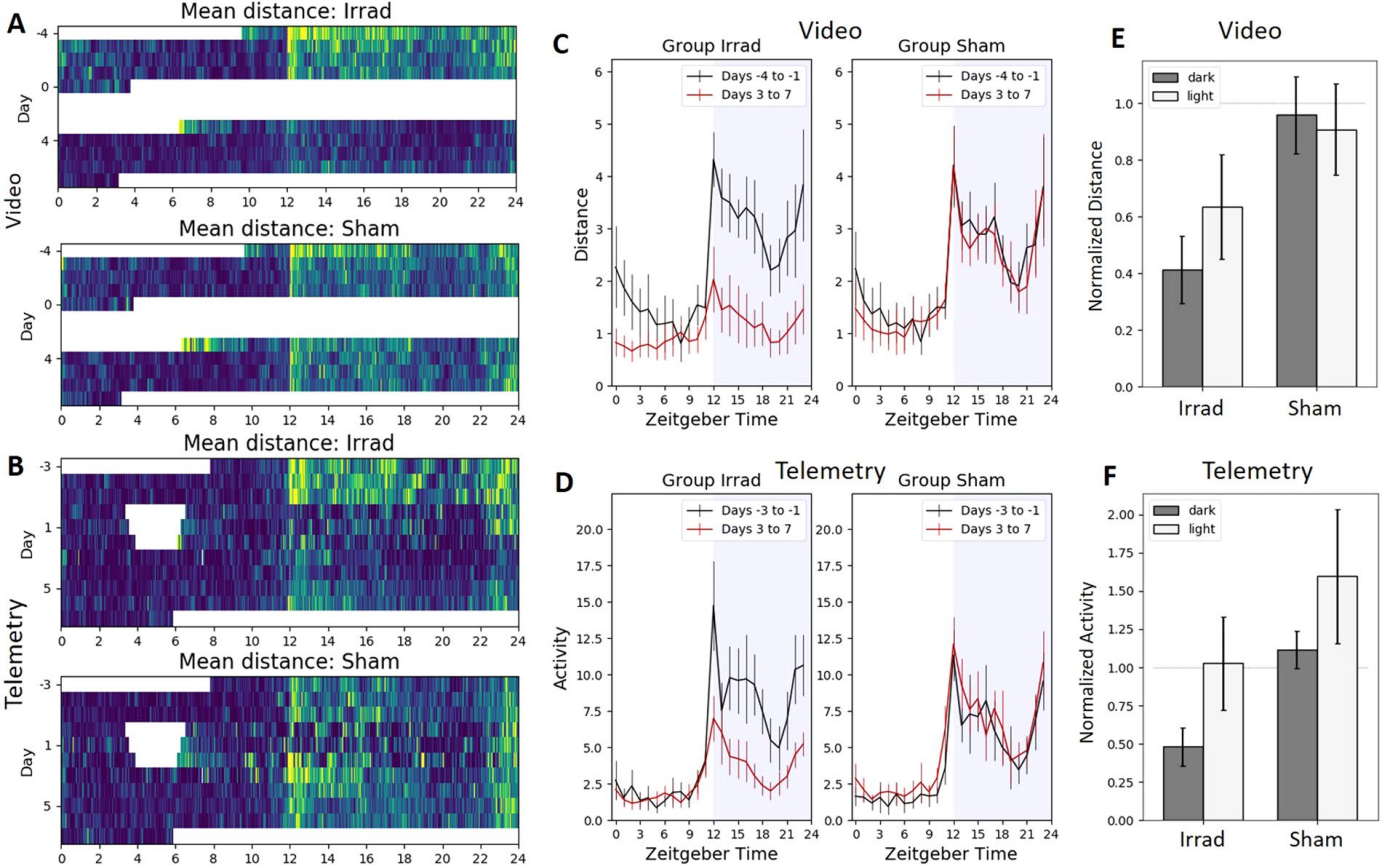
Circadian home cage activity patterns. Zeitgeber time = 0 when the lights are turned on at 6am. (**A**–**B**) Color-coded activity for each minute of video (**A**) and telemetry (**B**) recording, with brighter colors corresponding to more running wheel activity. (**C**) Video-recorded home cage activity averaged for every hour across four days of baseline and four days of post-irradiation recording. After irradiation, the irradiated group ran significantly less during the dark (one-tailed Bonferroni-corrected paired t-test, t_8_ = 9.50, p < 10^−4^) and the light (t_8_ = 3.96, p = 0.0042). (**D**) Telemetry-recorded home cage activity averaged for every hour across three days of baseline and four days of post-irradiation recording. After irradiation, the irradiated group ran significantly less during the dark (one-tailed Bonferroni-corrected paired t-test, t_9_ = 5.89, p < 10^−3^) but not during the light (t_9_ = 0.77, p = 0.46). (**E**) Video-recorded activity averaged for the 12-hour light period and the 12-hour dark period across four days of baseline and four days of post-irradiation recording. For video, two-way ANOVA showed significant effects of irradiation (F_1,38_ = 32.08, p < 10^−5^) and a significant interaction between irradiation and time period (F_1,38_ = 5.84, p = 0.02). Bonferroni-corrected p-values for two-tailed t-tests: Irrad dark vs light: t_17_ = 3.64, p = 0.008. Sham dark vs light: t_17_ = 0.52, p = 0.61. Dark, irrad vs sham: t_17_ = 6.54, p < 10^−4^. Light, irrad vs sham: t_17_ = 2.07, p = 0.21. (**F**) Telemetry-recorded activity averaged for the 12-hour light period and the 12-hour dark period across three days of baseline and four days of post-irradiation recording. For telemetry, there was a significant effect of irradiation (F_1,38_ = 21.36, p < 10^−4^) but no significant interaction between irradiation and time period (F_1,38_ = 0.06, p = 0.80). Bonferroni-corrected p-values for two-tailed t-tests: Irrad dark vs light: t_17_ = 3.48, p = 0.011. Sham dark vs light: t_17_ = 2.24, p = 0.16. Dark, irrad vs sham: t_17_ = 7.66, p < 10^−5^. Light, irrad vs sham: t_17_ = 2.31, p = 0.14.

Whether monitored by video (Fig. 6A) or telemetry (Fig. 6B), home cage activity occurred to some degree during both the light and dark periods. This contrasts with running wheels, which showed very little light period activity. After irradiation, both methods showed a large drop in physical activity during the dark cycle in the irradiated group but not in the sham group. Video tracking (Fig. 6C) showed that prior to irradiation, home cage activity would continue to some degree after the lights were turned on at 6am, and then very slowly subside over the course of the day. This was no longer the case after animals were irradiated; activity then dropped to minimal values within an hour or two. For video recordings, the decrease in activity after animals were irradiated was significant in both the dark period (p < 10^−4^) and the light period (p = 0.0042). Surprisingly, telemetry recordings showed different behavior than the video recordings during light period, with home cage activity dropping precipitously within an hour after the lights turned on even before irradiation (Fig. 6D). Telemetry recordings reported a significant post-irradiation decrease in dark period activity (p < 10^−3^) but not in light period activity (p = 0.46). Two-way ANOVA (Fig. 6C,F) showed significant effects of irradiation (p < 10^−3^) for both methods, but a significant interaction between irradiation and time period was found only for video monitoring (p = 0.02 for video, p = 0.8 for telemetry).

### Motor Screens

**Figure 7:**
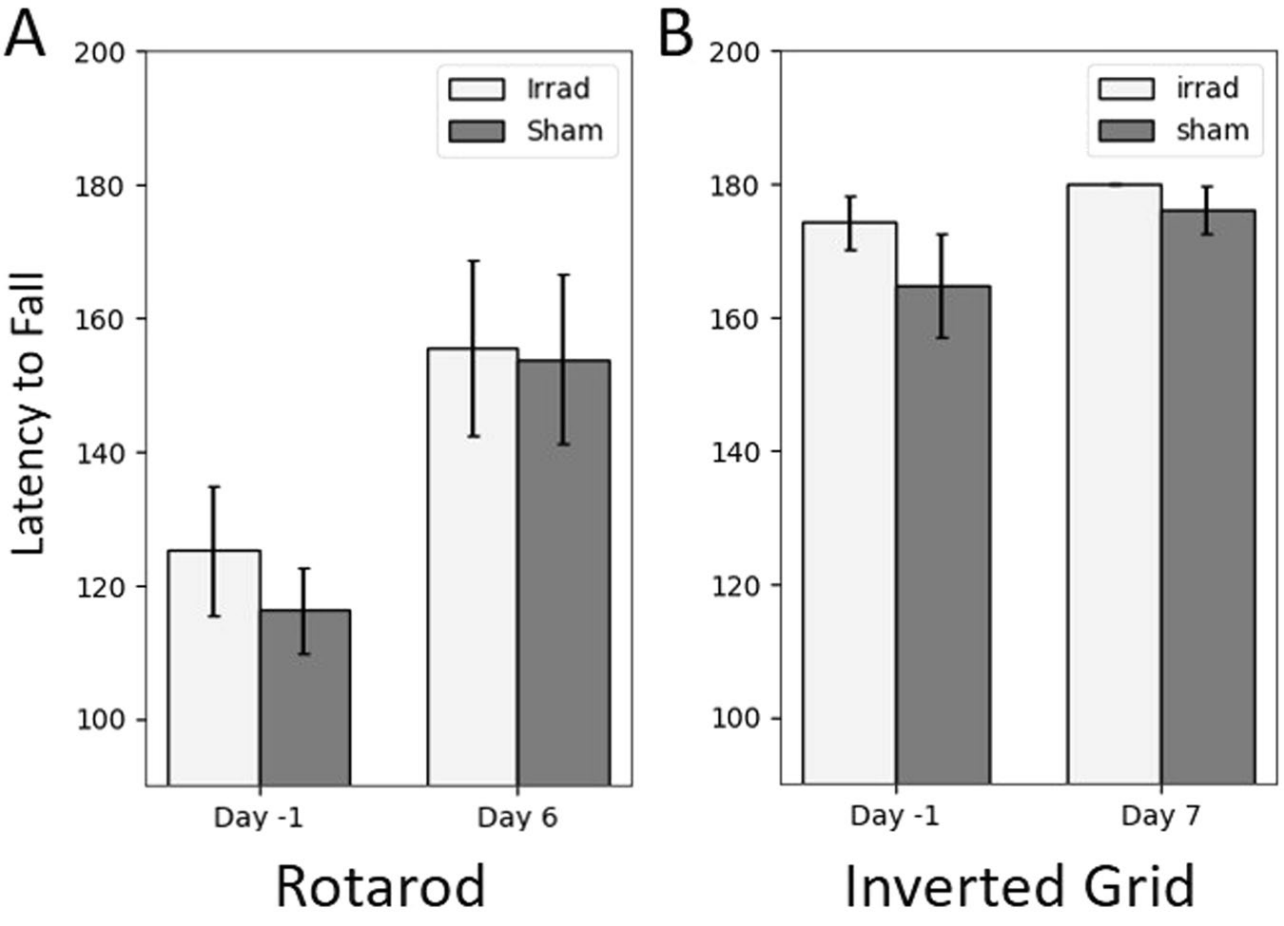
Mice were tested on either the inverted grid suspension test or the rotarod test both before and after irradiation. (**A**) The groups did not show a significant difference in performance before (two-tailed t-test, t_13_ = 0.74, p = 0.47) or after (two-tailed t-test, t_13_ = 0.09, p = 0.93) irradiation. (**B**) The groups performed similarly on the inverted grid suspension test, though performance was likely at ceiling. Error bars are standard error of the mean.

We hypothesized that changes in physical activity were not due to fatigue-like effects, but to deficits in the motor system. To test this hypothesis, we performed a pair of behavioral screens after irradiation (Fig. 7A,B). The rotarod test did not show any difference between groups either before (t_13_ = 0.74, p = 0.47) or after (t_13_ = 0.09, p = 0.93) irradiation, as determined by two-tailed t-tests. The inverted grid did not show differences between groups, but performance in the test was likely near ceiling both before and especially after irradiation, since the majority of mice maintained their grip for the full 180 seconds. The results from these experiments suggest that changes in daily physical activity after irradiation were not due to gross motor deficits.

### Body Weight

**Figure 8:**
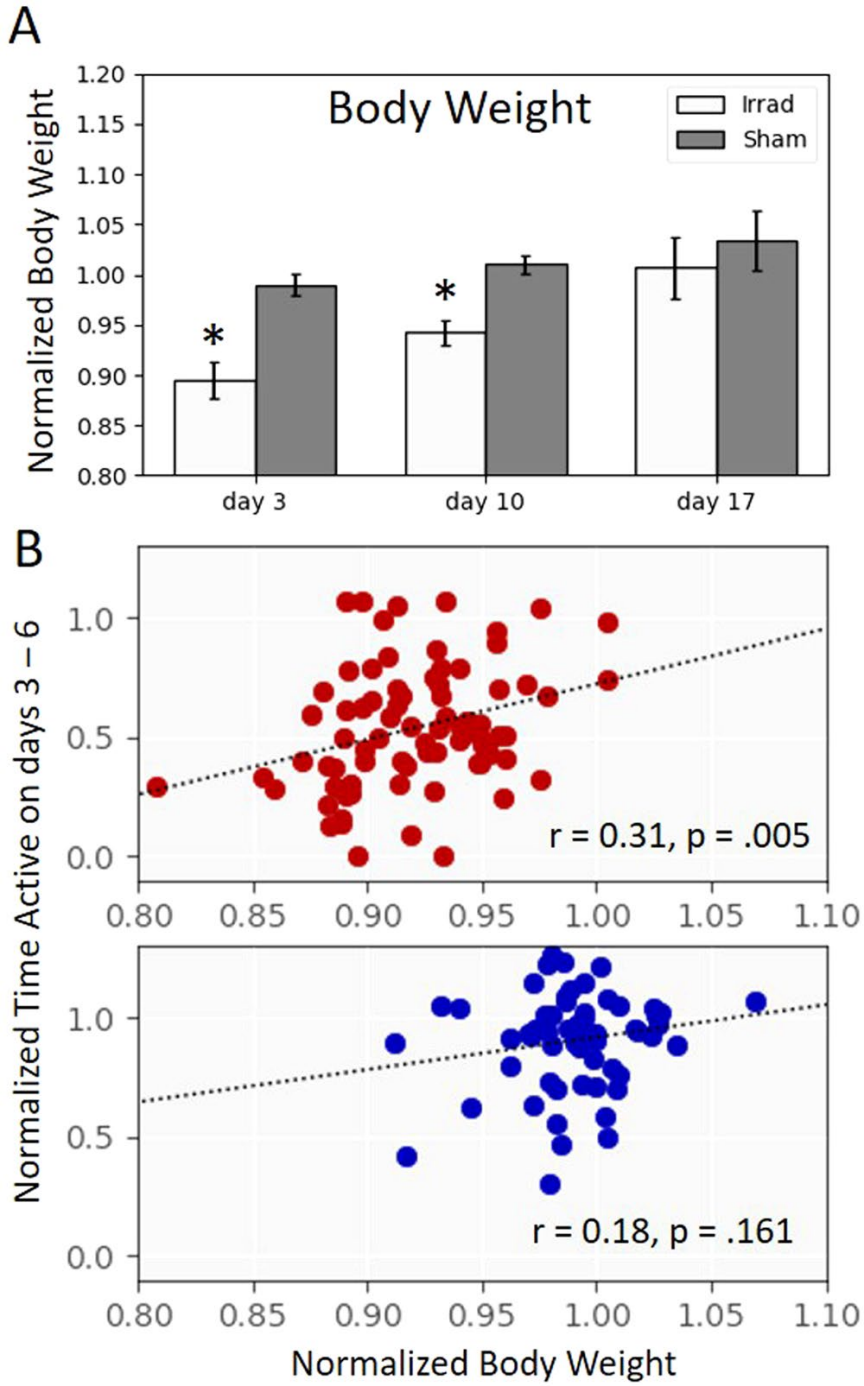
(**A**) Body weights in the irradiated group, normalized to baseline, were significantly lower than sham at day 3 (t_50_ = 10.2, p < 10^−13^) and day 10 (t_148_ = 8.39, p < 10^−13^), but not at day 17 (t_15_ = 1.60, p = 0.12). Error bars are 95% confidence intervals. (**B**) A post-irradiation decrease in body weight significantly correlated with the time active on running wheels on the four days immediately after irradiation for the irradiated group (top, r = 0.31, p = 0.005, n = 81) but not the sham group (bottom, r = 0.18, p = 0.161, n = 63).

The average body weight prior to irradiation was 22.9 ± 1.74 g (mean ± standard deviation). We found that body weight decreased immediately after a mouse went through the three-day irradiation procedure (Fig. 8A). Two-tailed t-tests determined that body weights in the irradiated group were significantly lower than sham at day 3 (t_50_ = 10.2, p < 10^−13^) and day 10 (t_148_ = 8.39, p < 10^−13^), but not at day 17 (t_15_ = 1.60, p = 0.12). The loss of body weight was significantly correlated with time active on the running wheels (Fig. 8B) for the irradiated group (r = 0.31, p = 0.005) but not the sham group (r = 0.18, p = 0.161). This suggests that the irradiation-induced fatigue may be accompanied by decreased food consumption.

## Discussion

The main goal of this study was to evaluate different passive measures of fatigue in mice. We induced a fatigue-like behavior using three days of peripheral irradiation, and in different sets of mice we measured running wheel activity, home cage activity using video, and home cage activity using telemetry. All these methods involve passive recording and, unlike alternate methods of measuring fatigue behaviors like open field or treadmills, the measures we used do not interfere with the normal, voluntary activity of the mice. Our study found specific strengths and weakness for each of these passive measures that can be useful in selecting outcomes to evaluate fatigue-like behavior.

Radiation induced a clear reduction in average daily running wheel activity, which became particularly evident when measuring “time active” on the wheels as opposed to total distance (number of wheel revolutions). The time active measure showed lower variability than the distance measure because of fluctuations in running speed that affect the measured distance. When evaluating fatigue-like behavior, investigators may find “time active” to be the more stable measure on a daily basis, particularly when looking at 1-minute intervals like we did. This idea may be supported by recent results testing chemotherapy-related fatigue, in which the time on the running wheel may appear to be more stable^14^, though calculated wheel time using 1-hour intervals. However, another study looking at post-exercise fatigue did not appear to find much difference between the two measures^21^.

In our circadian data, we saw a characteristic shift of running wheel activity to be more concentrated in the earlier hours of darkness after irradiation (Fig. 5A,B), similar to changes that have been observed in mouse models of chemotherapy^12^. This late activity reduction may be due to animals getting tired sooner while exercising on the running wheels, because we did not see the same change in the home cage activity data (Fig. 6A,B). One potential downside of using running wheels is that we occasionally observed mice (5 of the 32 in the sham group) that spontaneously reduced or completely stopped their use of running wheels with no clear reason, even after we double-checked proper function of the running wheel system and examined the health of the mice. Because of this experience, we advise that measurement of fatigue-like behavior using running wheels should incorporate a large sample size or be combined with other complementary methods like home cage activity monitoring.

The running wheels by themselves capture no information outside of wheel rotation, and only describe events during roughly half of the day. We found that if left undisturbed, mice rarely use running wheels during the light period^23^. However, it’s important to consider that running wheels measure a behavior that is different from home cage activity, where wheel usage depends on a mouse’s propensity to voluntarily exercise. Running wheel totals may decrease when animals become easily exhausted, whereas home cage activity may never push animals to the point of exhaustion. Mice find wheel running rewarding^26^, although the reasons and motivation for wheel running are not simple to explain^27,28^. Nonetheless, if interested in the motivational aspect of fatigue, one could argue that wheel running is a more important measure. Additionally, there is evidence that the presence of running wheels can “enrich” the environment and reverse some negative behavioral changes induced by single-housing the mice^29,30^. A final advantage of running wheels is that running wheels are overall easier to implement. They don’t require device implantation surgery and the data analysis is considerably more straight-forward than with video monitoring.

Video home cage monitoring reported the largest effect of irradiation on average daily activity (Fig. 4G) and was able to separate irradiated from sham groups based on a simple threshold of activity (Fig. 4B), and therefore seems like an ideal method for measuring fatigue-like behavior in mice. Activity at baseline showed a clear circadian pattern, with elevated activity during the dark period and a slow trailing off of activity during the light period (Fig. 6A,B). Irradiation resulted in a drop of activity across the entirety of the dark period and much of the light period as well. In fact, it was the only method that showed a large reduction of activity during the light period (Fig. 6C). The main drawback to video monitoring is its relative difficulty; 24-hour videos are large data files and time-consuming to analyze. In contrast to hour 24-hour per day video monitoring, other published studies of home cage video monitoring for fatigue have used shorter recording durations^7,8,18^, and only one found an effect with their fatigue model^18^.

Measurement by telemetry gives a less quantitative description of physical activity; while video provides a total distance travelled by the mouse, telemetry is a cruder measure of the motion of an implanted device relative to an external receiver. Nonetheless, telemetry reported a clear behavioral phenotype (Fig. 3B) with an effect size similar to running wheels (Fig. 4G), and a similar change in the circadian pattern of activity (Fig. 6D–F). Day-to-day, average daily telemetry measurements seemed less consistent than with video. After irradiation, the sham group recorded by video consistently showed an average close to the baseline (Fig. 3A), whereas the telemetry recordings showed daily averages up to 130% of baseline (Fig. 3B). Individual mice also showed a wider range of activity changes measured by telemetry relative to video tracking (Fig. 4C,B). Another published study using telemetry to characterize chemotherapy-induced fatigue measured a robust effect over five days of recording, though in that study it was not as effective as running wheels^11^.

In addition to the three main measurements, we performed a pair of screens for motor deficits, because irradiation-related damage to muscles or nerves could also cause a reduction in physical activity. In both the rotarod and inverted grid tests, we found no evidence of any change in performance after irradiation, though in the inverted grid, most of the mice were able to maintain their grip for the full 180 seconds; hence, their performance was likely at ceiling. Nonetheless, we concluded that the fatigue-like behavior we observed in our irradiated mice is unlikely to be caused by any neuromuscular conditions.

We did find a positive correlation between changes in body weight and running wheel activity (Fig. 8B). Reduced wheel running should itself is likely not the cause of reduced body weight, because a reduced body weight is more commonly associated with increased wheel running^26^. It is therefore likely that changes in food consumption are contributing to a change in body weight, and it is possible they contribute to the fatigue-like behavior as well.

### Applicability to other fatigue measures

There are couple limitations to this study that are worth pointing out. First, mice recovering from surgical implantation of telemetry devices experienced a longer “acclimation” phase (Fig. 1B) than mice without the devices. As a result, measurements using telemetry were in mice that were one to two weeks older than mice in the other groups. There is little evidence for whether this difference in age would affect radiation-induced fatigue, but the possibility should be acknowledged. Second, the cages used for each type of measurement were of different sizes, and this could potentially affect behavior. The PhenoTyper cages we used for video monitoring are slightly larger than the cages used for running wheels, which are in turn slightly larger than the cages used for telemetry measurements. Because the metal running wheels we used seemed to interfere with telemetry recordings, further experiments would benefit from recording video and telemetry in the same cages with the same mice. Limitations in equipment and space available to us made this impractical for this study. Nonetheless, while each method of measuring activity was on different mice in different environments, changes in activity levels both within groups (baseline vs post-irradiation) and across groups (irradiation vs sham) should make the results generalizable to some degree.

When choosing a method for measuring fatigue, it is important to consider what other information may be in the collected data. With both methods of home cage monitoring, video and telemetry, we have looked only at relatively simple physical activity measures. However, video recordings also include information about an animal’s location, specific behaviors (e.g., grooming), and interactions with the home cage environment (e.g., food and water access). Telemetry implants can include channels for measuring for other variables such as heart rate and blood pressure, which can be correlated with physical activity data. It is also important to note that this study only investigated behavioral changes as averages across days or within days. However, there may well be important information contained in the distance and speed measurements when observed on a time scale shorter than one day.

In conclusion, we found that running wheels and home cage monitoring are viable methods for measuring fatigue-like behavior in mice, and that video tracking is particularly effective. Future investigations looking at biology of fatigue or efficacy of anti-fatigue therapies should consider using variables that are generated from these sensitive passive measures as study outcomes.

## Methods

### Ethics

This study was approved by the National Heart Lung and Blood Institute (NHLBI) Animal Care and Use Committee of the National Institutes of Health (NIH), Bethesda, Maryland, USA. All investigators taking part in animal handling and measurement of study outcomes were properly trained by the NIH Office of Animal Care and Use and the NHLBI Murine Phenotyping Core. All aspects of animal testing, housing, and environmental conditions used in this study were in compliance with The Guide for the Care and Use of Laboratory Animals^31^.

### Animals

Male C57Bl/6 mice were ordered from Charles River Laboratories (Frederick MD) and were 6–9 weeks old at the beginning of each study. Mice were given ad libitum access to food and water and were individually housed on a 12:12 hour light-dark cycle throughout all studies. Tails were tattooed for identification, and mice received three days of gentle handling by experimenters before procedures began. Mice received daily visual health inspection and were removed from study if any health problems were apparent. Cages were cleaned once per week.

For fatigue monitoring, mice were assigned into three testing groups: 72 for running wheel activity, 20 for video home cage activity, and 22 for telemetry recording. In each of these testing group, half of the animals received irradiation and the other half received sham irradiation. For motor testing, a group of 16 mice housed with running wheels were designated for the inverted grid suspension test, and a separate group of 19 mice in regular home cages underwent the rotarod test. The main experiment design is displayed in Fig. 1 and described in each testing method below. The experimental sample sizes were determined by space and time constraints and not by statistical methods. Experimenters were not blinded to group, but the collection of physical activity data was automated.

### Irradiation

Mice were assigned to irradiated or sham groups so that body weights were evenly distributed between groups; grouping was otherwise random. Mice in the sham group underwent the same procedure as mice in the irradiation group, except the irradiator was not turned on. On each day of irradiation, mice were weighed and then anesthetized with an intraperitoneal injection of ketamine & xylazine (100 & 10 mg/kg, respectively). Mice were irradiated in a GammaCell 40 Irradiator (Best Theratronics, Ontario, Canada). Custom-made lead shielding targeted the irradiation to the lower abdomen and upper thighs, with dosimetry showing approximately 97% of the radiation blocked by the shielding. The lower abdominal radiation dose was 8 Gy per day (at a dose rate of roughly 1 Gy/min) for three consecutive days. Immediately following irradiation, mice were placed in home cages above heating pads until recovered from anesthesia, which lasted for about 45 minutes after the injection. Mice were able to recover from three consecutive days of anesthesia and this repeated dose of peripheral irradiation with no overt physical or behavioral alterations. This method was described in more detail in our previous papers^16,17^.

### Voluntary Wheel Running Activity (VWRA)

Mice were housed in cages with a running wheel (Lafayette Neuroscience, Indiana, USA) with the following dimensions: 33 × 19 × 19.5 cm (length × width × height), which recorded wheel rotation in one-minute intervals. After one week acclimating to the animal facility in standard plastic home cages, mice were transferred into running wheel cages where they stayed for 1–2 weeks before irradiation. Mice were removed from their running wheel cages during the three days of irradiation and placed back in running wheel cages the day after. Mice that did not use the running wheels were removed from the study. Data were collected by the Lafayette Running Wheel software and analyzed using Excel (Microsoft) or custom Python code.

### Video Home Cage Activity

Animals were tracked in PhenoTyper cages using Ethovision software, both by Noldus (Wageningen, The Netherlands). The PhenoTyper cages had interior dimensions of 29.5 × 29.5 × 33.5 cm (length × width × height) and did not have running wheels. After 2–3 weeks acclimating to the animal facility in standard plastic home cages, mice activities were recorded 24 hours per day by a video camera mounted at the top of each cage for at least four days prior to and at least four days immediately after irradiation. Mice were removed from the PhenoTyper cages during the three days of irradiation and placed back into the PhenoTyper cages the day after. Data (total distance traveled in the cage) was exported and analyzed using custom Python code.

### Telemetry Home Cage Activity

After one week acclimating to the animal facility in standard plastic home cages with dimensions of 30.5 × 19 × 11.5 cm (length × width × height), mice were surgically implanted with a telemetry device (DSI ETA-F10) that monitored physical activity. Mice were anesthetized with isoflurane, and the telemetry device was placed in the lower right abdominal cavity through an incision in the lower-left chest. Mice recovered on heating pads with bupivacaine applied to the incision site. Half of the mice were implanted one day, with the other half on the following day. After 15–16 days of recovery from surgery, the home cages were transferred onto telemetry receiver pads for the rest of the study. There were three days of recording, followed by three days of irradiation, then four more days of recording. Physical activity data were collected through the receiver underneath the home cage and recorded and analyzed using the Ponemah software (DSI, Minnesota, USA), Excel, and custom Python code.

### Rotarod

A Rotamex 5 rotarod (Columbus Instruments, Ohio, USA) was used to assess motor coordination and balance on a gradually accelerating rod. A soft disposable pad was placed on top of a thick foam pad for the mice to land on when they fell off the rod. Latency (seconds) for the mice to fall from the rod was recorded. Mice received two days of training and one day of testing on the three days before starting irradiation, and one day of testing on the fourth day after irradiation. We tested on the fourth day because our previous findings showed that fatigue-like behavior in mice was most pronounced 4–5 days after irradiation^16,17^. Data were analyzed using Excel.

### Inverted Grid Suspension Test

The inverted grid suspension test was used to assess grip strength and coordination in mice. Mice were placed on a grid or cage lid, which slowly turned over so they were suspended no higher than three feet above a padded surface, and the latency (seconds) to fall was recorded. Each test consisted of three 180-second trials per mouse, and mice were first tested the day before irradiation. For scheduling purposes and to ensure that the same research staff handles the mice and conduct the test, mice were again tested on the fifth day after irradiation. Data were analyzed using Excel.

### Statistical Analysis

All statistical calculations used α = 0.05. “Outlier” data points were removed from analysis if they were more than three standard deviations from the mean. Parametric tests were only performed after passing (p > 0.05) based on a Shapiro-Wilk normality test using either SPSS (for mixed-model ANOVA) or the scipy.stats.shapiro() function in Python for t-tests. T-tests were conducted using the scipy.stats.ttest_ind() function for Python, with Welch’s t-test used when samples had unequal variance as determined by Levene’s test (scipy.stats.levene()). Two-sample Kolmogorov-Smirnov (KS) tests were conducted using the scipy.stats.ks_2samp() function in Python. Correlations and their significance were calculated using the scipy.stats.pearsonr() function in Python. Two-way ANOVAs were conducted using the statsmodels.formula.api.ols() and statsmodels.stats.anova.anova_lm() functions in Python. Linear mixed-model ANOVA was conducted using SPSS; Levenes’ test for equality of variance provided non-significant values in all cases (p > 0.05), and within-subjects effects were calculated with Greenhouse-Geisser corrections when the sphericity assumption was violated according to Mauchly’s test. Bonferroni or Bonferroni-Holm corrections for multiple comparisons were implemented where indicated in the results section for each test.
